# Genomic analysis of stayability in Nellore cattle

**DOI:** 10.1371/journal.pone.0179076

**Published:** 2017-06-07

**Authors:** Daniela Barreto Amaral Teixeira, Gerardo Alves Fernandes Júnior, Danielly Beraldo dos Santos Silva, Raphael Bermal Costa, Luciana Takada, Daniel Gustavo Mansan Gordo, Tiago Bresolin, Roberto Carvalheiro, Fernando Baldi, Lucia Galvão de Albuquerque

**Affiliations:** 1 Faculdade de Ciências Agrárias e Veterinárias, UNESP, Jaboticabal, SP, Brazil; 2 Escola de Medicina Veterinária e Zootecnia, UFBA, Salvador, BA, Brazil; 3 CNPq Fellowship, Brasília, DF, Brazil; China Agricultural University, CHINA

## Abstract

Stayability, which can be defined as the probability of a cow calving at a certain age when given the opportunity, is an important reproductive trait in beef cattle because it is directly related to herd profitability. The objective of this study was to estimate genetic parameters and to identify possible genomic regions associated with the phenotypic expression of stayability in Nellore cows. The variance components were estimated by Bayesian inference using a threshold animal model that included the systematic effects of contemporary group and sexual precocity and the random effects of animal and residual. The SNP effects were estimated by the single-step genomic BLUP method using information of 2,838 animals (2,020 females and 930 sires) genotyped with the Illumina High-Density BeadChip Array (San Diego, CA, USA). The variance explained by windows formed by 200 consecutive SNPs was used to identify genomic regions of largest effect on the expression of stayability. The heritability was 0.11 ± 0.01 when *A* matrix (pedigree) was used and 0.14 ± 0.01 when *H* matrix (relationship matrix that combines pedigree information and SNP data) was used. A total of 147 candidate genes for stayability were identified on chromosomes 1, 2, 5, 6, 9 and 20 and on the X chromosome. New candidate regions for stayability were detected, most of them related to reproductive, immunological and central nervous system functions.

## Introduction

There is growing concern regarding the increase in the world population and the consequent increasing global demand for food. One solution to mitigate a possible food crisis is to increase animal productivity. According to FAO estimates, by 2050, Brazil will be responsible for providing 40% of the global food demand. The country currently possesses about 219 million cattle [1].

When it comes to increasing productivity in the agricultural sector, especially beef cattle, reproductive rates are the most important factors that need to be considered. In Nellore cattle, reproductive traits have been proved to be 4 to 13 times economically more important than growth traits as reported by [2]. According to [3], cows are the animal category that consumes most of the feed available for the herd. The maintenance of cows is therefore a major component of beef cattle production costs and these costs increase when the reproductive rates of the herd are low [4]. According to [5], the selection of replacement heifers is practically impossible in the case of low reproductive rates. It is therefore important that the cow remains in the herd until an economic return compatible with its rearing, breeding and maintenance costs is obtained [6, 7].

Female reproductive traits, which have until recently been little explored in beef cattle breeding programs because of the greater emphasis on the selection and evaluation of sires and because of their low heritabilities and difficult measurement, are gaining increasing interest. An economically important female reproductive trait is stayability (STAY), which is defined as the probability of a cow remaining in the herd until a specific age given the opportunity to reach this age [8]. According to [3], the inclusion of this trait in genetic evaluation programs allows to select sires that produce longer-living daughters.

STAY is a trait that is measured late in the life of an animal and is only evaluated in females. Males should be evaluated by progeny testing, which increases the generation interval. Moreover, this trait shows low heritability and is therefore not commonly used as a selection criterion in beef cattle. Heritability values reported by [9] and [10] for STAY in Nellore cattle were 0.07 and 0.10, respectively.

The inclusion of single nucleotide polymorphisms (SNPs) in analyses has allowed more accurate genetic evaluations of different productive traits, with a special advantage for the evaluation of traits that are expressed late and that require the implementation of a progeny test for the evaluation of sires, as is the case of STAY [11]. A simple and practical approach to include SNPs in the genetic evaluation of a trait of interest is to implement the single-step GBLUP method (ssGBLUP) proposed by [12]. The main advantage of this method is the possibility to simultaneously combine in the analyses all information available, including phenotypes, pedigree information and SNP data. Using ssGBLUP, solutions of mixed model equations can be applied to a genome-wide association study (GWAS) to identify potential genomic regions involved in the phenotypic expression of the trait studied [13]. Some genes associated with reproductive traits in Zebu cattle have already been described by GWAS [14, 15, 16]. However, few studies on STAY in Nellore cattle have been published.

The objective of the present study was to identify and investigate genomic regions with the largest effect on the expression of STAY in Nellore cattle. The identification of these genomic regions will allow a better understanding and evaluation of this trait, in addition to identify candidate genes for future studies.

## Material and methods

This work was approved by the ethical committee on the use of animals (CEUA) of the School of Agricultural and Veterinarian Sciences (FCAV/UNESP). Protocol number 18340/16.

### Phenotypic data

The database used was obtained from the livestock archive of eight farms located in the following towns and states: Valparaíso/SP, Cotegipe/BA, Água Clara/MS, Itaquiraí/MS, Comodoro/MT, Jussara/MT, Tangará da Serra/MT, and Goianésia/GO. The farms to which these herds belong are dedicated to beef cattle farming and are part of the Aliança Nelore database, which combines data from the DeltaGen® and Paint® (CRV Lagoa) breeding programs.

The reproductive management adopted by the farms included in this study refers to two breeding seasons: a) an anticipated breeding season from February to April during which heifers at about 14 to 18 months of age are challenged for 60 days in order to identify sexually precocious animals; b) a breeding season that starts in November and lasts 90 days in which all females of breeding age of the herd participate to the breeding season. Heifers that do not conceive in the anticipated breeding season have another opportunity in the second season. Females, heifers and cows that do not conceive in the normal breeding season (November) are culled.

Records from 127,561 cows born between 1980 and 2009 were used. Stayability was defined as a binary trait, attributing value 1 (failure) or value 2 (success) to each female. The starting point adopted for STAY was the age at first calving (AFC), including females with AFC of more than 22 months and less than 45 months in the analysis. In this study, success was defined when the female remained productive in the herd for a period of 65 months or longer, as suggested by [17].

The contemporary groups were defined as year, farm and season of birth of the cow. Season of birth was classified as dry (March to August) and rainy (September to February). Contemporary groups without variability and those with fewer than four animals were excluded from the analyses.

The precocity of each cow was calculated as a function of AFC, attributing score 1 (precocious) to females with AFC ≤ 31 months and 2 (non-precocious) to females with AFC > 31 months.

After data editing, 96,811 animals remained for further analysis. Among these, 20,757 (21.44%) were precocious and 25,532 (26.37%) received value 2 (success) for STAY (Table 1). The pedigree file used in the analysis contained 405,776 animals.

**Table 1:** Descriptive statistics of the dataset.

	Score	Frequence	Proportion %
**STAY**	1	71,279	73.63
	2	25,532	26.37
**Precocity**	1	20,757	21.44
	2	76,054	78.56

STAY = Stayability

### Genotypic data

The genotype file contained information for 2,020 females and 930 sires from the Aliança Nelore database genotyped with the Illumina High-Density BeadChip Array (San Diego, CA, USA) that contains 777,962 SNPs. SNPs located on autosomes and on the X chromosome were considered. For analysis, the genotype of the female sex chromosome was coded as 0 for females homozygous for the first allele (A), as 1 for heterozygous females, and as 2 for those homozygous for the second allele (B). On the other hand, SNPs of the male X chromosome were coded as 0 or 2, where 0 = A_ and 2 B_. The same methodology was adopted by [18] in a GWAS of postpartum anestrus in Brahman and tropical beef cattle breeds.

The preGSf90 program [12] was used for quality control analysis of the genotypes, excluding SNPs with a MAF < 0.05 and a call rate < 0.90 from the analyses. After genotype quality control, the final dataset consisted of 2,838 animals and 463,345 SNPs.

### Statistical analysis

An additive threshold animal model including the systematic effects of contemporary group and precocity was used to obtain the solutions of mixed model equations. A generalized linear mixed model with a probit link function was fitted using the Thrgibbsf90 software [19]. The model can be written in matrix form as follows [20]:

η=Xβ+Zu+e

where *η* is the linear predictor; ***X*** and ***Z*** are incidence matrices of the systematic (contemporary group and precocity) and random effects, respectively; *β* is the vector of fixed effects; *u* is the vector of additive effect of the animal assuming $u∼N(0,\;{H\sigma }_{u}^{2})$, where ***H*** is the relationship matrix that combines pedigree information (matrix **A**) and SNP data (matrix **G**), as described by [21], and $\sigma_{u}^{2}$ is the additive genetic variance; *e* is the vector of residual effect assuming $e∼N(0,I\sigma_{e}^{2})$, where ***I*** is an identity matrix and $\sigma_{e}^{2}$ is the variance of residual effects.

In this model, the inverse of the numerator relationship matrix, ***A***^-1^, was replaced with ***H***^-1^, as described by [21]:

$$H^{-1}=A^{-1}+\left|\begin{array}{cc} 0 & 0\\ 0 & G^{-1}-A_{22}^{-1} \end{array}\right|$$

where $A_{22}^{-1}$ is the inverse of the additive relationship matrix for genotyped animals and ***G***^-1^ is the inverse of the genomic relationship matrix. Matrix ***G*** was obtained as follows [22]:

G=ZDZ′q

where ***Z*** is the genotype matrix adjusted for allele frequencies; ***D*** is a diagonal matrix containing weights for the effects of SNPs (initially, ***D*** *=* ***I***), and *q* is a normalization factor. According to [23], these weights can be obtained by ensuring that the average diagonal in ***G*** is close to $A_{22}^{-1}$.

A total of 500,000 Gibbs chains were generated in the analyses. One sample was removed every 10 iterations, considering a burn-in period of 30,000 iterations. Convergence was analyzed with the Postgibbsf90 software [19] and the Coda package of the R program [24].

The SNP effects were computed iteratively using the postGSf90 program according to the procedure described by [13]. The equation used to calculate the SNP effects can be written in matrix form as:

$${\hat{u}}_{s}=DZ^{\prime }[ZDZ\prime {]}^{-1}{\hat{u}}_{g}$$

where *û*_*s*_ is the vector with the effect of each SNP; ***D*** is a diagonal matrix containing weights for the SNP effect; ***Z*** is the genotype matrix, and *û*_*g*_ is the vector with the estimated breeding values for genotyped animals. In this study, *û*_*g*_ was computed once and *û*_*s*_ was obtained considering two iterations, in which matrix ***D*** was equal to an identity matrix in the first iteration, while in the second one it became a diagonal matrix containing the weights calculated in the previous iteration. The objective of these iterations is to increase the weight of SNPs with large effects and to reduce the weight of those with small effects [25].

The 10 windows of 200 SNPs based on the highest additive genetic variance were considered. The genes were identified using the NCBI Map Viewer tool (www.ncbi.nlm.nih.gov/mapview/)—Bos taurus UMD 3.1.1, Annotation Release 104 (accession GCF 000003055.6). Functional analysis of the mapped genes was performed using the DAVID v6.7 software (https://david.ncifcrf.gov/). ClueGo, a Cytoscape plug-in (http://apps.cytoscape.org/apps/cluego), was used to construct a network of functionally grouped genes based on biological terms in order to identify associations with biological processes.

## Results and discussion

The estimated heritability (h^2^) for STAY was higher when the genomic information was included in the analyses through matrix *H* (Table 2). The estimates were 0.14 (0.01) and 0.11 (0.01) with *H* matrix and *A* matrix, respectively. The combination of genomic and pedigree information allowed to capture a higher proportion of the additive genetic variability of STAY in the population studied. This result can be explained by the fact that 39% of the animals were offspring of multiple sires. These findings agree with [26] who found that the heritabilities for carcass traits in Nellore cattle estimated with the ssGBLUP method were higher than those obtained with BLUP and that the addition of genomic information to *A* matrix resulted in greater additive genetic variance for the traits with a smaller number of observations.

**Table 2:** Variance components and genetic parameters for stayability in Nellore cattle using two different relationship matrices (A or H).

Parameters	A	H
Additive genetic variance ($\sigma_{a}^{2}$)	0.12 ± 0.02	0.16 ± 0.02
Residual variance ($\sigma_{e}^{2}$)	1.00 ± 0.01	1.00 ± 0.01
Heritability (h^2^)	0.11 ± 0.01	0.14 ± 0.01
h^2^ high density interval (HPD)	0.08 to 0.13	0.11 to 0.17

A = Pedigree-based relationship matrix; H = Combined pedigree-genomic relationship matrix

We found no studies in the literature estimating heritability for STAY using *H* matrix. However, the present results corroborate those of other studies using *A* matrix. [27] estimated heritabilities of 0.12 to 0.36 for STAY from 2 to 8 years in Canadian Simmental cattle. In Nellore cattle, [10] and [28] obtained heritabilities of 0.10 and 0.19, respectively.

**Fig 1:**
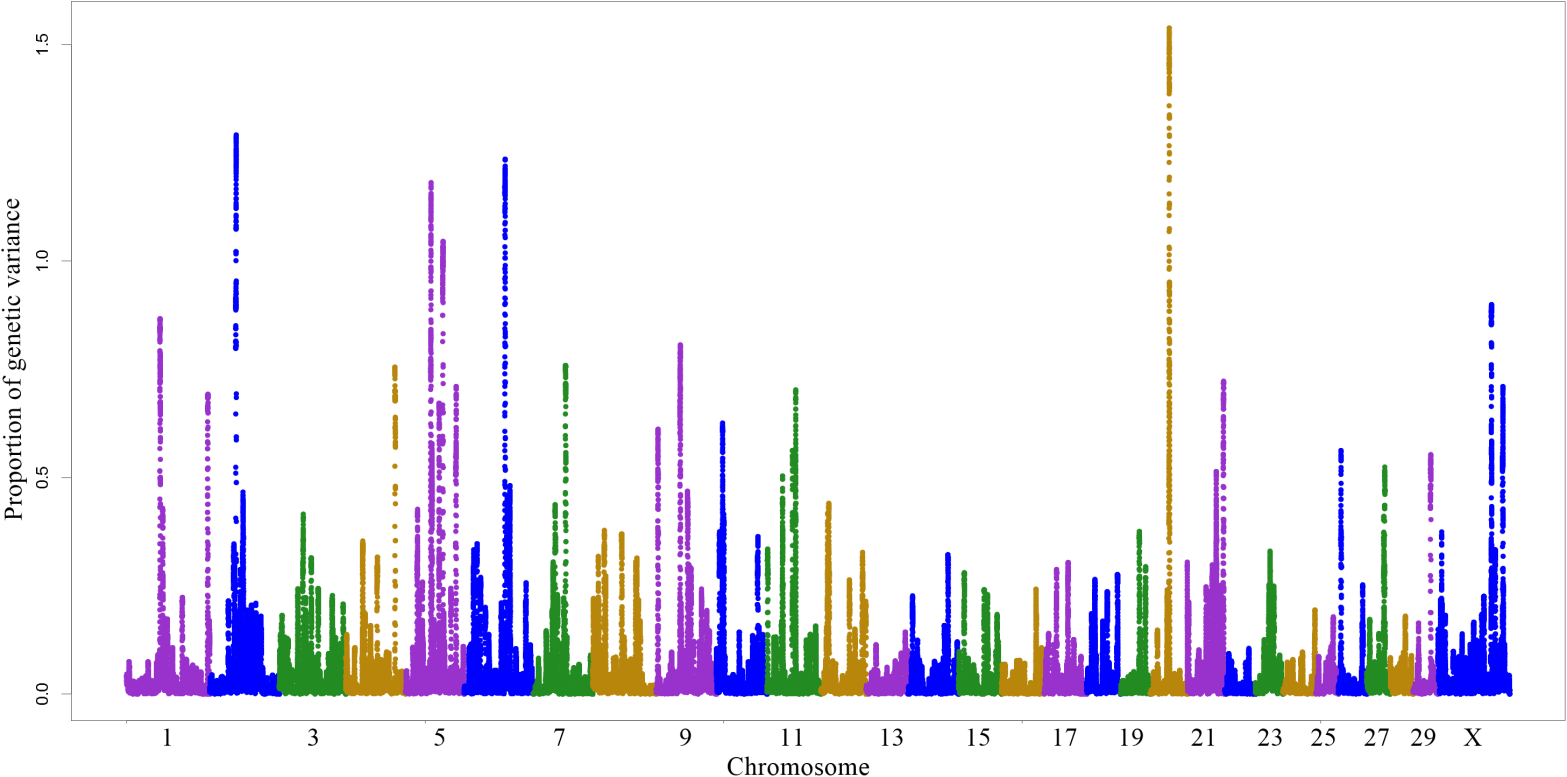
Manhattan plot of the variance explained by windows formed by 200 consecutive SNPs for stayability in Nellore cattle.

The 10 windows that explained a higher proportion of additive genetic variance were located within or near 147 genes distributed across six autosomes (BTA 1, 2, 5, 6, 9 and 20) and on the X chromosome (Fig 1), corresponding to 10.28% of the total additive genetic variance (Table 3). Of these 147 genes, 78 encode proteins; four are transcribed to microRNAs that play a role in the regulation of other genes; four are transcribed to transporter RNA; one gene encodes the open reading frame (ORF) of a region of a given protein, and 60 are LOCs that were divided into protein-coding genes, pseudogenes, unknown non-coding RNAs, and proteins uncharacterized in cattle.

**Table 3:** Chromosome (Chr), position, candidate genes and proportion of variance (Var) explained by the top 10 SNP windows with higher effects for stayability.

Chr	Position	Genes	Var
1	63294181–64393269	LOC104970860, LOC104970861, LOC104970862, LOC104970863, LOC104970864, IGSF11	0.87
2	49170368–50130815	LOC104971217, LOC786829, LOC526380	1.29
5	47361341–49420148	LOC100337478, TRNAC-GCA, LOC525592, LOC104969784, HELB, LOC783726, IRAK3, TRNAK-CUU, TMBIM4, LLPH, LOC100848387, HMGA2, LOC101905641, MIR763, LOC101905683, MSRB3, LOC104972440, LEMD3, WIF1, MIR2429, LOC101905989, TBC1D30, LOC101906182, GNS, RASSF3, RASSF3	1.18
5	76437773–77662501	ALG10, SYT10, PKP2, TRNAC-GCA, YARS2, DNM1L, LOC101907810, FGD4	1.04
5	102131720–104053171	CLEC4D, CLEC4E, LOC786242, CD163, LOC101908561, LOC101907409, LOC751811, LOC104972550, WC-7, LOC751788, LOC104972549, LOC751789, WC1-12, LOC100335428, LOC100336766, LOC786796, LOC100335470, LOC104972551, WC1-10, LOC100299671, WC1.3, WC1, WC1-2, WC1-8, LOC540180, LOC100139350, PEX5, LOC104972559, CLSTN3, RBP5, C1RL, LOC786586, LOC507706, LOC783686, LOC101902570, C1R, C1S, LPCAT3, EMG1, PHB2, LOC104972560, MIR141, MIR200C, PTPN6, C5H12orf57, ATN1, ENO2, LRRC23, LOC104972561, SPSB2, TPI1, USP5, CDCA3, GNB3, P3H3, GPR162, CD4, LAG3,PTMS	0.71
6	71300961–72320057	PDGFRA, LOC100296974, LOC104968886, LOC100296505, KIT, LOC104972758, LOC104972757, LOC104972756, LOC104972755, LOC104972754, LOC100138563, KDR, LOC104968888, LOC101906337	1.23
9	40739084–41780597	FIG4,AK9,ZBTB24,MICAL1,SMPD2,PPIL6,CD164, SESN1,ARMC2,CEP57L1,CCDC162P,LOC104969539	0.81
20	39442753–40219577	RAI14, C1QTNF3, AMACR, SLC45A2, RXFP3, ADAMTS12, LOC101902009, LOC104975280	1.54
X	125360732–125958768	ARX, POLA1, LOC100296181, PCYT1B, LOC101905398, PDK3	0.90
X	141657572–142411095	LOC101904216, TRNAD-GUC, MSL3, LOC104970633, ARHGAP6	0.71

On BTA1, some LOC regions and the IGSF11 gene (immunoglobulin family) were identified. No studies are available for *Bos taurus*, but this gene is homologous in humans and mice. According to [29], the gene is related to the central nervous system development which regulates the function of different hormones, including sex hormones such as LH, FSH and prolactin. Only as yet uncharacterized LOC regions were identified on BTA2.

Three of the 10 largest windows of genetic variance were identified on BTA5, which corresponded to about 3% of the total variance explained by the markers (Table 3). Regions responsible for the expression of reproductive traits (quantitative trait loci, QTLs) such ovulation and twinning rate have been identified on this chromosome [30, 31, 32, 33]. Daetwyler et al. [30] and Cruickshank et al. [34] found that the window of 45 to 50 Mb on BTA5 is associated with AFC and twinning rate in cattle. These results confirm that this region is involved in the expression of reproductive traits in cattle and may directly affect STAY of the animal.

Cai et al. [35] described a polymorphism in the HELB gene (also located in window 3, BTA5) that leads to lower levels of helicase B activity, affecting the proliferation of Sertoli cells. These cells are found in the testis where they control the maturation and migration of germ cells. As an indirect consequence of the HELB gene polymorphism, the proliferation of Sertoli cells would be reduced, as would be the levels of inhibin. The latter is a hormone produced by the testes and ovarian follicles that acts as a regulator of spermatogenesis and ovulation.

Regions on BTA5 have also been associated with productive and reproductive traits in *Bos indicus* and *Bos taurus* [36] that are directly influenced by the immune system. Some genes of the WC1 family have also been mapped to the window 5 of BTA5. This multigene family plays a role in the modulation of immune responses, being involved in signaling through γδ T-cell receptors [37]. According to these authors, γδ T cells play an important role in the immune response because of their capacity to produce IFN-γ and, thus, to trigger responses against antigens.

**Fig 2:**
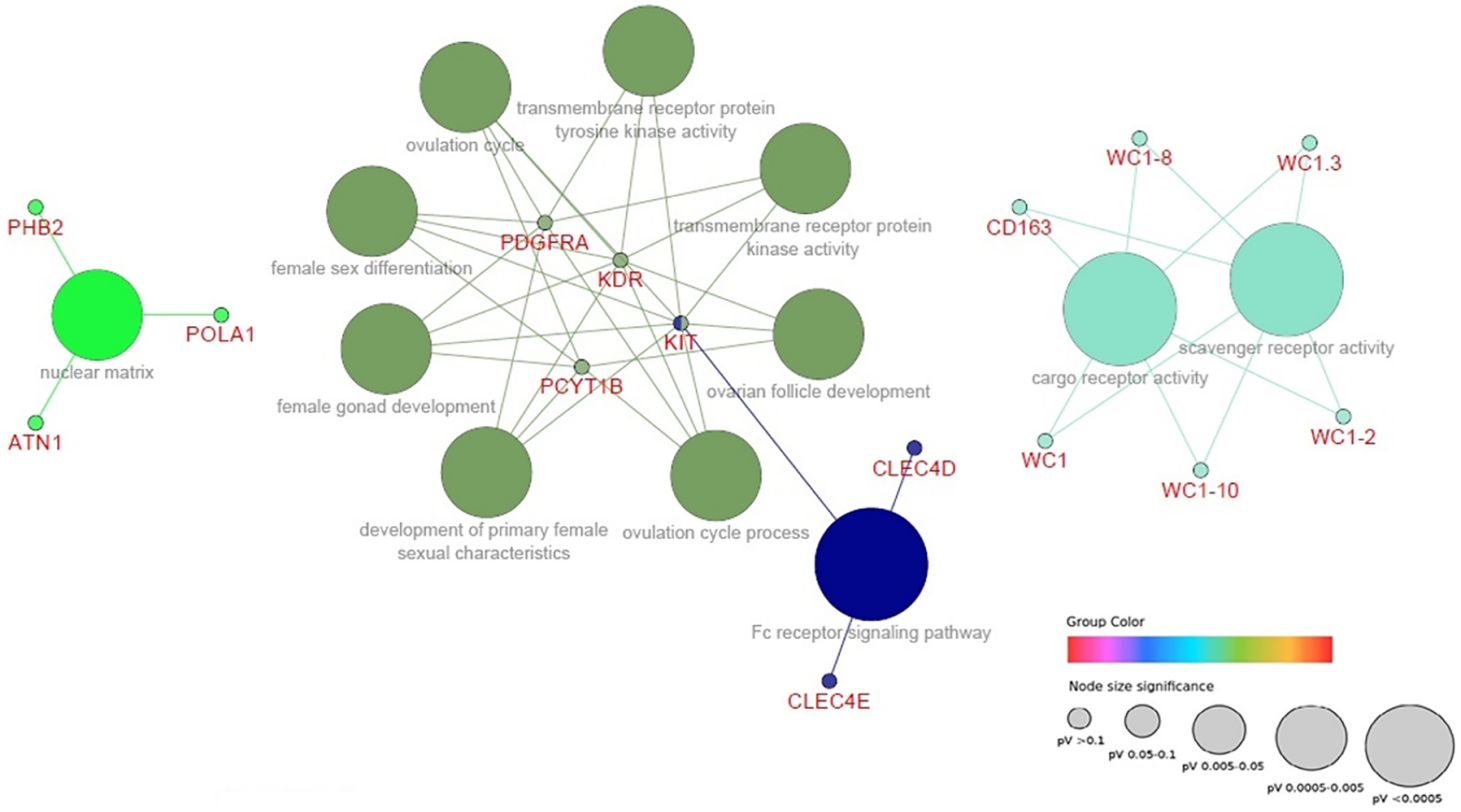
Gene network linked to biological process related to stayability in Nellore cattle. The size of nodes represents the statistical significance of the terms. (P-value adjusted to Bonferroni statistics).

Investigation of the metabolic processes in which the genes mapped to the 10 largest windows of variance are involved (DAVID v6.7 software) showed an enriched pathway (KEGG database) with genes related to reproductive traits associated with STAY. This pathway is related to endocytosis and contains three genes (KDR, PDGFRA and KIT) located in the window of BTA6. These genes play an important role in cellular processes such as cell regulation, proliferation and differentiation. As can be seen in Fig 2, the KDR, PDGFRA and KIT genes were functionally grouped and are related to the reproductive system, forming a cluster of tyrosine kinase receptors.

The PDGFRA gene on BTA6 is related to the PDGF gene and both PDGFRs. These genes are expressed in the ovary of different species, influencing the transition from primordial to primary follicles [38] and stimulating cell proliferation [39] and follicular development [40]. In the mouse ovary, PDGFRA is found in luteinizing cells derived from the theca of the developing corpus luteum. In an *in vitro* study, Woad et al. [41] showed that PDGF activity regulates the formation of the luteal endothelial network in bovine ovaries.

Another expressed gene found on BTA6 is KIT. This gene controls key cellular processes, including the migration, proliferation, differentiation and survival of cells. The KIT gene, together with its cleavage products, is found in somatic, germ and gonadal cells of both sexes. KITL/KIT signaling promotes the growth, maturation and survival of germ cells in the gonads. In the ovary, the expression of KIT in the cellular stroma accompanies the development of the thecal layer around advanced follicles [42].

The CD164 gene on chromosome 9 was studied by [43] and their results suggest that an increased expression of the gene is associated with the progression of ovary cancer in humans and mice.

The RAI14 gene on chromosome 20 has been associated with spermatogenesis in mice. According to [44], RAI14 is a candidate gene for mediating the effect of the NR2F2 gene on ovarian cancer in humans.

The PDK3 gene located on the X chromosome was studied by [45], who concluded that the gene is involved in the maturation and meiosis of mouse oocytes. Regueira et al. [46] highlighted the importance of this gene for the hormonal regulation of Sertoli cell production. The ARX gene was also found on the X chromosome, which is homologous in humans and mice. This gene provides instructions for the production of a protein that regulates the activity of other genes. The ARX gene acts during early embryo development to control the formation of various structures of the body. In the developing brain, the ARX protein is involved in the movement (migration) and communication of nerve cells (neurons). At least 30 mutations in the ARX gene can cause X-linked lissencephaly with abnormal genitalia in humans [47].

## Conclusions

The results of the present study revealed a list of 147 candidate genes for STAY in Nellore cattle. New candidate regions for STAY were detected, most of them related to reproductive, immunological and nervous system functions.
